# A Novel, Extremely Elongated, and Endocellular Bacterial Symbiont Supports Cuticle Formation of a Grain Pest Beetle

**DOI:** 10.1128/mBio.01482-17

**Published:** 2017-09-26

**Authors:** Bin Hirota, Genta Okude, Hisashi Anbutsu, Ryo Futahashi, Minoru Moriyama, Xian-Ying Meng, Naruo Nikoh, Ryuichi Koga, Takema Fukatsu

**Affiliations:** a National Institute of Advanced Industrial Science and Technology (AIST), Tsukuba, Japan; b Department of Biological Sciences, Graduate School of Science, the University of Tokyo, Tokyo, Japan; c Computational Bio Big-Data Open Innovation Laboratory, National Institute of Advanced Industrial Science and Technology (AIST), Tokyo, Japan; d Department of Liberal Arts, the Open University of Japan, Chiba, Japan; e Graduate School of Life and Environmental Sciences, University of Tsukuba, Tsukuba, Japan; University of Hawaii at Manoa

**Keywords:** bacterial symbiont, bacteriocyte, bacteriome, *Bacteroidetes*, cuticle formation, *Oryzaephilus surinamensis*, saw-toothed grain beetle, Silvanidae, stored-product pest

## Abstract

The saw-toothed grain beetle, *Oryzaephilus surinamensis* (Silvanidae), is a cosmopolitan stored-product pest. Early studies on *O. surinamensis* in the 1930s described the presence of peculiar bacteriomes harboring endosymbiotic bacteria in the abdomen. Since then, however, the microbiological nature of the symbiont has been elusive. Here we investigated the endosymbiotic system of *O. surinamensis* in detail. In the abdomen of adults, pupae, and larvae, four oval bacteriomes were consistently identified, whose cytoplasm was full of extremely elongated tubular bacterial cells several micrometers wide and several hundred micrometers long. Molecular phylogenetic analysis identified the symbiont as a member of the *Bacteroidetes*, in which the symbiont was the most closely related to the endosymbiont of a grain pest beetle, *Rhyzopertha dominica* (Bostrichidae). The symbiont was detected in developing embryos, corroborating vertical symbiont transmission through host generations. The symbiont gene showed AT-biased nucleotide composition and accelerated molecular evolution, plausibly reflecting degenerative evolution of the symbiont genome. When the symbiont infection was experimentally removed, the aposymbiotic insects grew and reproduced normally, but exhibited a slightly but significantly more reddish cuticle and lighter body mass. These results indicate that the symbiont of *O. surinamensis* is not essential for the host’s growth and reproduction but contributes to the host’s cuticle formation. Symbiont genome sequencing and detailed comparison of fitness parameters between symbiotic and aposymbiotic insects under various environmental conditions will provide further insights into the symbiont’s biological roles for the stored-product pest.

## IMPORTANCE

Some beetles notorious as stored-product pests possess well-developed symbiotic organs called bacteriomes for harboring specific symbiotic bacteria, although their biological roles have been poorly understood. Here we report a peculiar endosymbiotic system of a grain pest beetle, *Oryzaephilus surinamensis*, in which four oval bacteriomes in the abdomen are full of extremely elongated tubular bacterial cells. Experimental symbiont elimination did not hinder the host’s growth and reproduction, but resulted in emergence of reddish beetles, uncovering the symbiont’s involvement in host’s cuticle formation. We speculate that the extremely elongated symbiont cell morphology might be due to the degenerative symbiont genome deficient in bacterial cell division and/or cell wall formation, which highlights an evolutionary consequence of intimate host-symbiont coevolution.

## INTRODUCTION

Many insects host symbiotic bacteria in their gut, body cavity, or cells (1–3). Some bacterial symbionts are indispensable for growth, survival, and reproduction of their insect hosts via synthesis and supply of essential nutrients (4, 5), whereas other bacterial symbionts are dispensable, entailing either positive, neutral, or negative fitness consequences depending on environmental conditions, for their insect hosts as facultative microbial associates (6, 7). Among them, the most highly integrated host-symbiont associations are found in insects with specialized symbiotic organs, designated bacteriomes, consisting of host cells for harboring the symbiotic bacteria endocellularly, called bacteriocytes (1, 8, 9). Following early histological descriptions (reviewed in reference 1), bacteriome-associated endosymbiotic bacteria have been microbiologically characterized using modern molecular techniques from diverse insect orders, including the Hemiptera (aphids, coccids, whiteflies, leafhoppers, cicadas, stinkbugs, etc.) (10–16), the Diptera (tsetse flies, bat flies, louse flies, etc.) (17–19), the Blattodea (cockroaches, wood roaches, etc.) (20, 21), the Phthiraptera (sucking lice, chewing lice, etc.) (22, 23), and the Coleoptera (beetles). For a long time, the Curculionidae (weevils) and the Chrysomelidae (leaf beetles) have been the only beetle families whose bacteriome-associated endosymbiotic bacteria were microbiologically identified. As for weevils, an ancient gammaproteobacterial endosymbiont clade, *Nardonella* spp., found in diverse weevils (24, 25), a gammaproteobacterial endosymbiont, *Sodalis pierantonius*, that presumably replaced the original *Nardonella* endosymbiont in the lineage of grain weevils of the genus *Sitophilus* (26, 27), and another gammaproteobacterial endosymbiont, *Curculioniphilus buchneri*, that also replaced the original *Nardonella* endosymbiont in the lineage of acorn weevils of the genus *Curculio* and allied species (28–30), have been described. As for leaf beetles, a gammaproteobacterial endosymbiont clade, *Macropleicola* spp., associated with reed beetles of the subfamily Donaciinae (31–33), and several other gammaproteobacterial endosymbionts (34, 35) have been reported.

Beetles, characterized by their thick and hard exoskeleton, represent the majority of the insect species ever described (36). Some beetles are known to infest stored cereals and other durable commodities, being notorious as stored-product pests (37, 38). Possibly relevant to their peculiar ecology of continuously living on monotonous and non-fresh food sources, stored-product pest beetles are often associated with symbiotic microorganisms, including the *Sitophilus* grain weevils (Curculionidae) with bacteriome-harbored gammaproteobacterium *Sodalis pierantonius* (26, 27), the cigarette beetle *Lasioderma serricorne* and the drugstore beetle *Stegobium paniceum* (Anobiidae) with yeast-like fungal symbionts *Symbiotaphrina* spp. in the gut-associated symbiotic organs both endocellularly and extracellularly (39–41), and the lesser grain borer, *Rhyzopertha dominica* (Bostrichidae), whose bacteriome-associated endosymbiont was recently identified as belonging to the *Bacteroidetes* (42). Considering their easy rearing, experimental tractability, and economic importance, identification and understanding of biological roles of the bacteriome-associated endosymbionts in such stored-product pests are of general relevance to both basic and applied aspects of entomology and microbiology.

**FIG 1:**
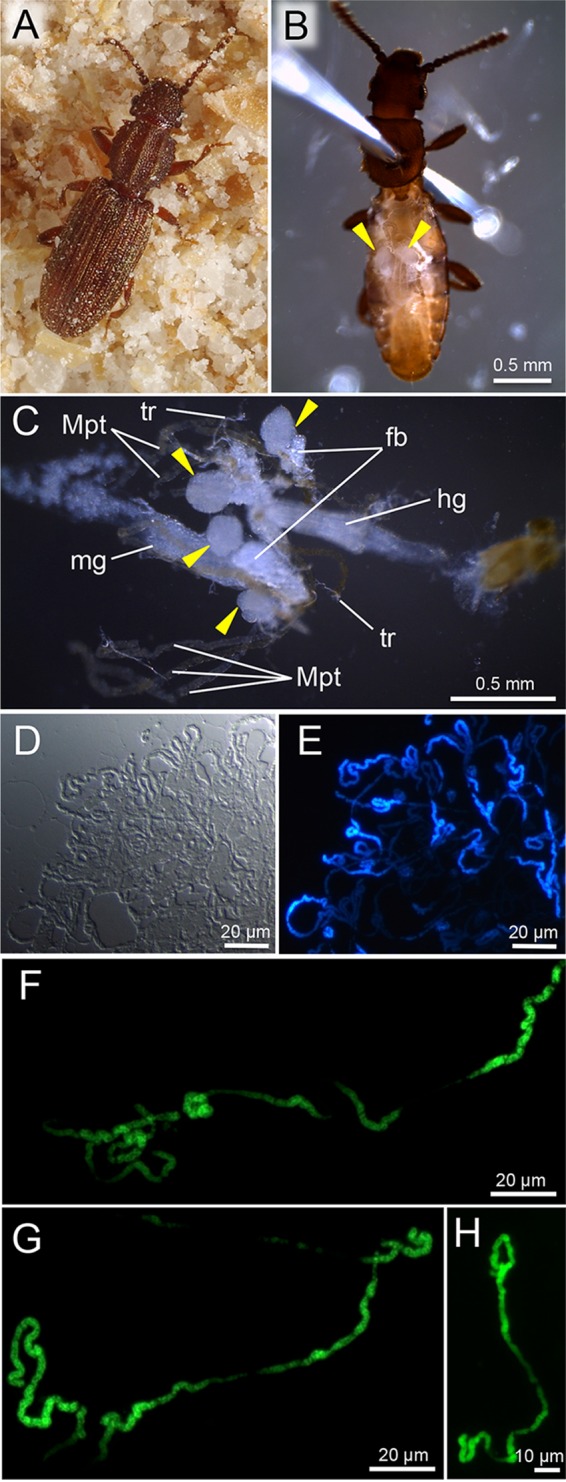
Bacteriomes and symbiotic bacteria of *O. surinamensis*. (A) Adult insect reared on whole-wheat flour. (B) Dissected adult insect, in which oval translucent bacteriomes are seen in the abdomen (arrowheads). (C) Internal organs dissected from an adult insect, in which four bacteriomes are highlighted (arrowheads). Note that reproductive tissues, fat body, and tracheae are mostly removed. Abbreviations: fb, fat body; hg, hindgut; mg, midgut; Mpt, Malpighian tubule; tr, trachea. (D and E) Differential interference microscopic image (D) and epifluorescence microscopic image (E) of symbiotic bacteria released from a dissected adult bacteriome. (F and G) Extremely elongated individual symbiont cells from dissected adult bacteriomes. (H) Elongated symbiont cell from a dissected pupal bacteriome. Bacterial DNA is stained with 4′,6-diamidino-2-phenylindole in panel E and SYTOX Green in panels F to H.

The saw-toothed grain beetle, *Oryzaephilus surinamensis* (Coleoptera: Silvanidae) (Fig. 1A), is a cosmopolitan stored-product pest (43, 44). The peculiar formation of the symbiotic system in *O. surinamensis*, consisting of four oval bacteriomes harboring a dense bacterial population and located in the abdomen, was first described histologically in the 1930s (45). Subsequent experimental works reported that symbiont elimination did not hinder growth and reproduction of *O. surinamensis* (46, 47), posing an enigma regarding the biological role of the symbiont for the insect host. Since then, however, the bacteriome-associated endosymbiosis in *O. surinamensis* has been totally untouched, leaving the understanding of the microbiological nature of the symbiont still elusive.

In this study, we investigated the endosymbiotic system of *O. surinamensis* to elucidate the microbiological nature of the symbiont, the fine structure of the symbiont, localization of the symbiont in the host tissues and cells, conditions for experimental symbiont elimination, and effects of symbiont removal on host phenotypes, by using modern molecular, histological, and morphometric techniques. We identified the symbiont as a novel bacterial lineage belonging to the *Bacteroidetes*, confirmed the histological descriptions in old literature, including endosymbiotic constitution with four oval bacteriomes and transovarial symbiont transmission to eggs, and newly discovered that the symbiont cells are extraordinarily elongated in shape and that the symbiont is certainly not essential for the host’s growth and reproduction but contributes to the host’s cuticle formation.

## RESULTS

### Extremely elongated symbiotic bacteria in bacteriomes.

When we dissected adult insects of the laboratory strain of *O. surinamensis*, translucent oval tissues were found in the abdomen (Fig. 1B), which were closely associated with Malpighian tubules, fat bodies, and tracheae (Fig. 1C). Smearing of the dissected tissues on a glass slide with DNA-binding fluorochromes visualized a dense population of tubular bacterial cells (Fig. 1D and E), verifying the tissues as constituting the bacteriomes of *O. surinamensis*. Strikingly, the symbiont cells were extremely elongated: in contrast to the cell width of a few micrometers, the cell length often reached some hundred micrometers, although the exact cell size was difficult to evaluate (Fig. 1F to H). The early histological study described that the symbiotic bacteria of *O. surinamensis* achieve their maximal length of 60 to 70 μm (45), but our observation unveiled much longer symbiont cell lengths than previously recognized.

### Bacterial 16S rRNA gene of the symbiont.

The dissected adult abdomens were subjected to DNA extraction, PCR, and cloning and sequencing of the bacterial 16S rRNA gene, which yielded a 1,499-bp nucleotide sequence (sequence accession no. LC314798). BLASTN searches of the DNA databases using the sequence as query identified 16S rRNA gene sequences of *Blattabacterium* spp., the endosymbiont clade associated with cockroaches (e.g., GenBank accession no. CP005488, CP003605, and AP014609), as the top hits. From adult insects of different sources collected at Shizuoka and Niigata, Japan, the same 16S rRNA gene sequences were obtained (sequence accession no. LC314799 and LC314800), indicating that a specific bacterial symbiont is associated with *O. surinamensis*.

### Phylogenetic placement of the symbiont.

**FIG 2:**
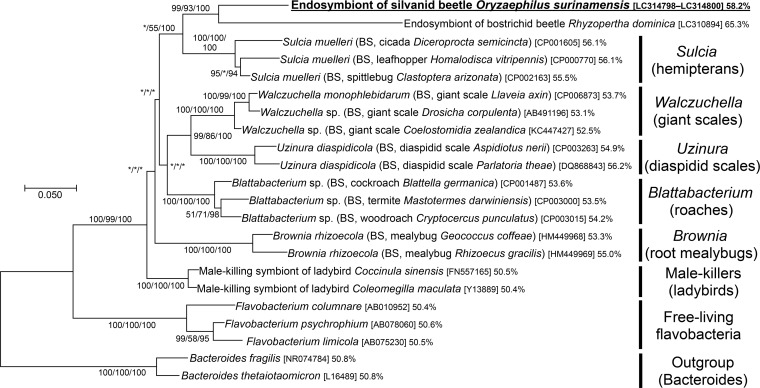
Phylogenetic placement of the symbiont of *O. surinamensis* in the *Bacteroidetes* based on 16S rRNA gene sequences. A maximum likelihood phylogeny inferred from 1,185 aligned nucleotide sites is shown. Statistical support values for each clade are shown in the order of bootstrap probability of the neighbor-joining analysis, bootstrap probability of the maximum likelihood analysis, and posterior probability of the Bayesian analysis from left to right, where asterisks indicate values less than 50%. Host-related information in parentheses, accession number in brackets, and percentage of AT content follow each bacterial taxon. “BS” indicates bacteriocyte-associated endosymbiont. The explanation for each major clade is depicted on the right side.

On the molecular phylogeny inferred from the 16S rRNA gene sequences, the symbiont of *O. surinamensis* was placed in the bacterial phylum *Bacteroidetes*, where the symbiont formed a distinct lineage clustering with endosymbiont clades associated with other insects, such as *Sulcia* of diverse hemipterans, *Walczuchella* of giant scale insects, *Uzinura* of diaspidid scale insects, *Blattabacterium* of cockroaches, *Brownia* of root mealybugs, etc. (Fig. 2). Notably, the symbiont of *O. surinamensis* (Silvanidae) was the most closely related to a recently reported, rosette-shaped bacterial endosymbiont of a stored-product pest, the lesser grain borer, *R. dominica* (Bostrichidae) (42) (Fig. 2).

### *In vivo* localization and fine structure of the symbiont.

**FIG 3:**
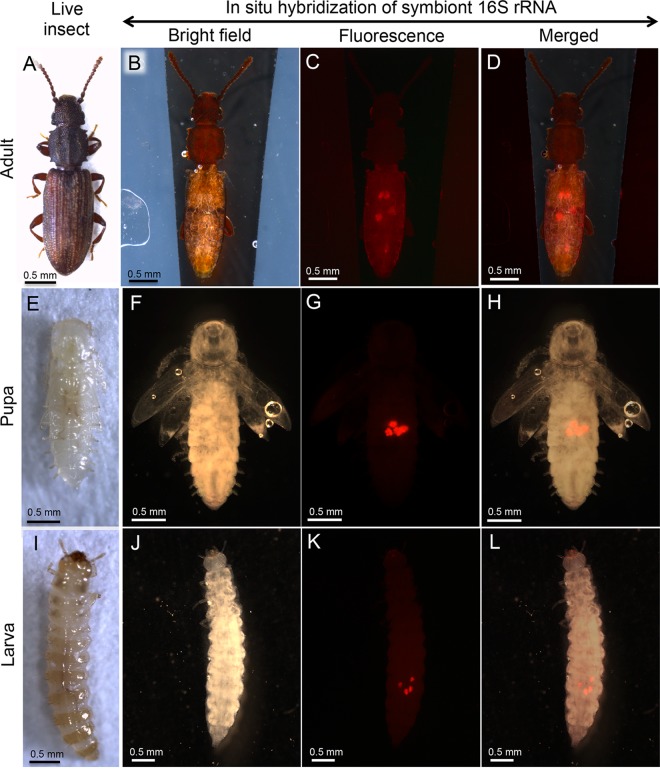
Localization of bacteriomes in adults, pupae, and larvae of *O. surinamensis*. (A to D) Adults. (E to H) Pupae. (I to L) Larvae. (A, E, and I) Live insect images. (B, F, and J) Bright-field images of the insects subjected to whole-mount *in situ* hybridization targeting 16S rRNA of the symbiont. (C, G, and K) Epifluorescence dissection microscopic images of the same insects, in which four bacteriomes in the abdomen are visualized in red. (D, H, and L) Merged images. In panels B to D, F to H, and J to L, the insect bodies were gently pressed by a coverslip to locate the four bacteriomes to the same focal plane. In panels B to D, thin silicon rubber plates for stabilizing the position of the insect body are seen on both sides.

**FIG 4:**
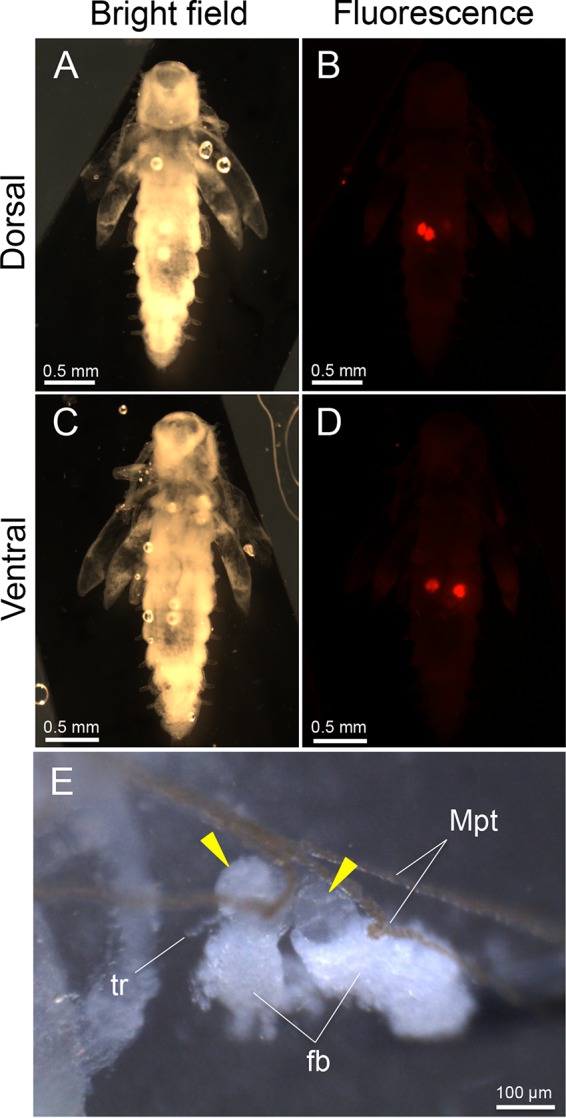
Localization and tissue construction of bacteriomes in pupae of *O. surinamensis*. (A) Bright-field image from the dorsal side. (B) Epifluorescence dissection microscopic image from the dorsal side, in which two dorsal bacteriomes are visualized in red by whole-mount *in situ* hybridization targeting 16S rRNA of the symbiont. (C) Bright-field image from the ventral side. (D) Epifluorescence dissection microscopic image from the ventral side, in which two ventral bacteriomes are seen in red. In panels A to D, different from Fig. 3F to H, the insect body was not pressed, thereby enabling the observation of the dorsal and ventral bacteriomes separately. (E) Magnified image of two dorsal bacteriomes (arrowheads) dissected from a pupa. Abbreviations: fb, fat body; Mpt, Malpighian tubule; tr, trachea.

**FIG 5:**
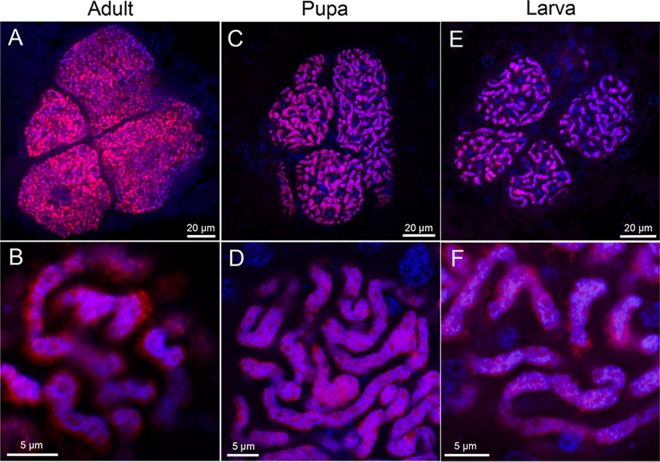
Laser confocal microscopic images of bacteriomes and symbiotic bacteria of *O. surinamensis*. (A and B) Adult bacteriome. (C and D) Pupal bacteriome. (E and F) Larval bacteriome. (A, C, and E) Images of dissected bacteriomes. (B, D, and F) Enlarged images of symbiotic bacteria in the cytoplasm of the bacteriome. Red signals represent 16S rRNA of the symbiont visualized by fluorescence *in situ* hybridization, whereas blue signals are due to DNA staining.

**FIG 6:**
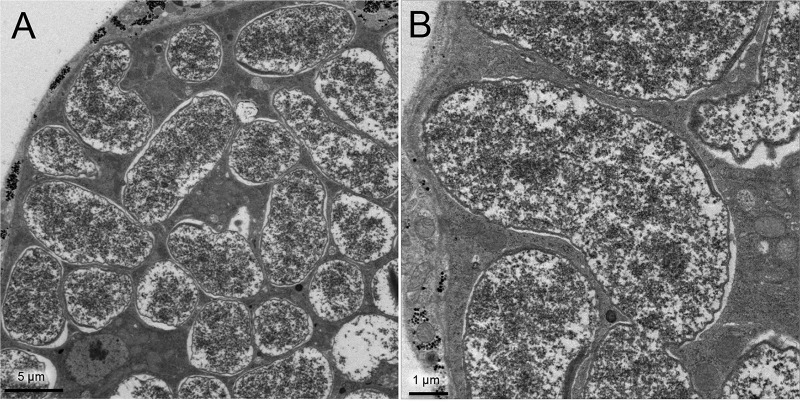
Fine structure of the symbiont of *O. surinamensis*. (A) Transmission electron microscopic image of a pupal bacteriocyte. (B) Magnified image of the symbiotic bacteria.

**FIG 7:**
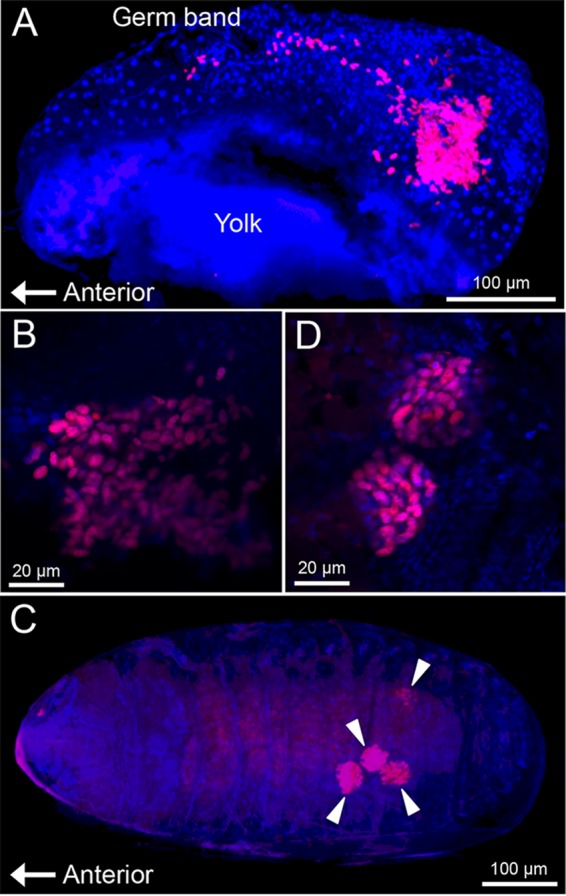
Localization of symbiotic bacteria in embryos of *O. surinamensis*. (A) Early embryo, in which a symbiont population in the germ band is seen in a posterior region. (B) Enlarged image of the bacterial population. (C) Late embryo, in which four primordial bacteriomes are seen in the abdomen (arrowheads). (D) Enlarged image of the primordial bacteriomes. Red signals represent 16S rRNA of the symbiont visualized by fluorescence *in situ* hybridization, whereas blue signals are due to DNA staining.

In order to visualize the symbiont localization *in vivo*, adults (Fig. 3A), pupae (Fig. 3E), and larvae (Fig. 3I) of *O. surinamensis* were subjected to whole-mount *in situ* hybridization targeting 16S rRNA of the symbiont. Four bacteriomes were consistently detected in the abdomen of adults (Fig. 3B to D), pupae (Fig. 3F to H), and larvae (Fig. 3J to L), of which two were situated dorsally (Fig. 4A and B) and the other two were located ventrally (Fig. 4C and D), in close association with fat body cells, tracheae, and Malpighian tubules (Fig. 1C; Fig. 4E). The peculiar formation of the symbiotic system is concordant with the previous histological descriptions of four oval bacteriomes in the abdomen of *O. surinamensis* (45, 46). Confocal fluorescence microscopy of the dissected bacteriomes of adults (Fig. 5A and B), pupae (Fig. 5C and D), and larvae (Fig. 5E and F) consistently visualized tubular bacterial cells filling the cytoplasm (Fig. 5). Transmission electron microscopy unveiled the fine structure of the symbiotic bacteria within the cytoplasm of the bacteriome (Fig. 6). In young embryos, a population of symbiotic bacteria was observed in a posterior region of the germ band (Fig. 7A and B). In mature embryos, four primordial bacteriomes were already formed in the abdomen (Fig. 7C and D). These observations favor the continuous host-symbiont association and the vertical symbiont transmission in *O. surinamensis* as described previously (45, 46).

### Molecular evolutionary aspects of the symbiont.

The AT content of 16S rRNA gene sequence of the symbiont of *O. surinamensis*, 58.2%, was higher than those of the other endosymbiont lineages in the *Bacteroidetes*, including *Sulcia* (55 to 56%), *Brownia* (53 to 55%), *Blattabacterium* (53 to 54%), and *Uzinura* (55 to 56%), and much higher than those of free-living *Flavobacteria* (50 to 51%) (Fig. 2). A relative rate test based on the 16S rRNA gene sequence showed that the evolutionary rate in the lineage of the symbiont of *O. surinamensis* was significantly higher than that in the lineage of free-living *Flavobacteria*, while being almost equivalent to those in the other endosymbiont lineages, like *Sulcia* and *Blattabacterium* (Table 1). These results suggest that the symbiont of *O. surinamensis* may have experienced reductive genome evolution like the other insect endosymbiont lineages in the *Bacteroidetes*. Notably, the symbiont of *O. surinamensis* exhibited remarkably lower AT content and a significantly slower molecular evolutionary rate than the symbiont of *R. dominica* (Table 1), suggesting the possibility that the symbiont genome reduction might have been less advanced in *O. surinamensis* than in *R. dominica*.

**TABLE 1:** Relative rate tests of 16S rRNA gene sequence of the symbiont of *Oryzaephilus surinamensis* in comparison with the symbiont of *Rhyzopertha dominica*, *Sulcia* symbionts, *Blattabacterium* symbionts, and allied free-living bacteria

Lineage 1	Lineage 2	Outgroup	*K*_1_^a^	*K*_2_^b^	*K*_1_ − *K*_2_	*K*_1_/*K*_2_	*P* value^c^
Symbiont of *O. surinamensis* (LC314798)	Symbiont of *R. dominica* (LC310894)	*Bacteroides fragilis* (NR_074784)	0.062	0.134	−0.072	0.46	6.4 × 10^−6^
Symbiont of *O. surinamensis* (LC314798)	*Sulcia* symbionts^d^	*Bacteroides fragilis* (NR_074784)	0.075	0.070	0.005	1.1	0.65
Symbiont of *O. surinamensis* (LC314798)	*Blattabacterium* symbionts^e^	*Bacteroides fragilis* (NR_074784)	0.068	0.051	0.017	1.3	0.12
Symbiont of *O. surinamensis* (LC314798)	*F. columnare* and *F. limicola*^f^	*Bacteroides fragilis* (NR_074784)	0.126	0.092	0.034	1.4	0.024

a Estimated mean distance between lineage 1 and the last common ancestor of lineages 1 and 2.

b Estimated mean distance between lineage 2 and the last common ancestor of lineages 1 and 2.

c *P* values were generated using the program RRTree (87). The analyses were performed using 1,425 aligned nucleotide sites of 16S rRNA gene sequences.

d Based on 16S rRNA sequences of *Sulcia* symbionts from *Homalodisca vitripennis* (CP000770), *Diceroprocta semicincta* (CP001605), and *Clastoptera arizonana* (CP002163).

e Based on  16S rRNA sequences of *Blattabacterium* symbionts from *Blattella germanica* (CP001487), *Mastotermes darwiniensis* (CP003000), and *Cryptocercus punctulatus* (CP003015).

f Based on 16S rRNA sequences of *Flavobacterium columnare* (AB010952) and *Flavobacterium limicola* (AB075230).

### Generation of an aposymbiotic host line by antibiotic treatment.

**FIG 8:**
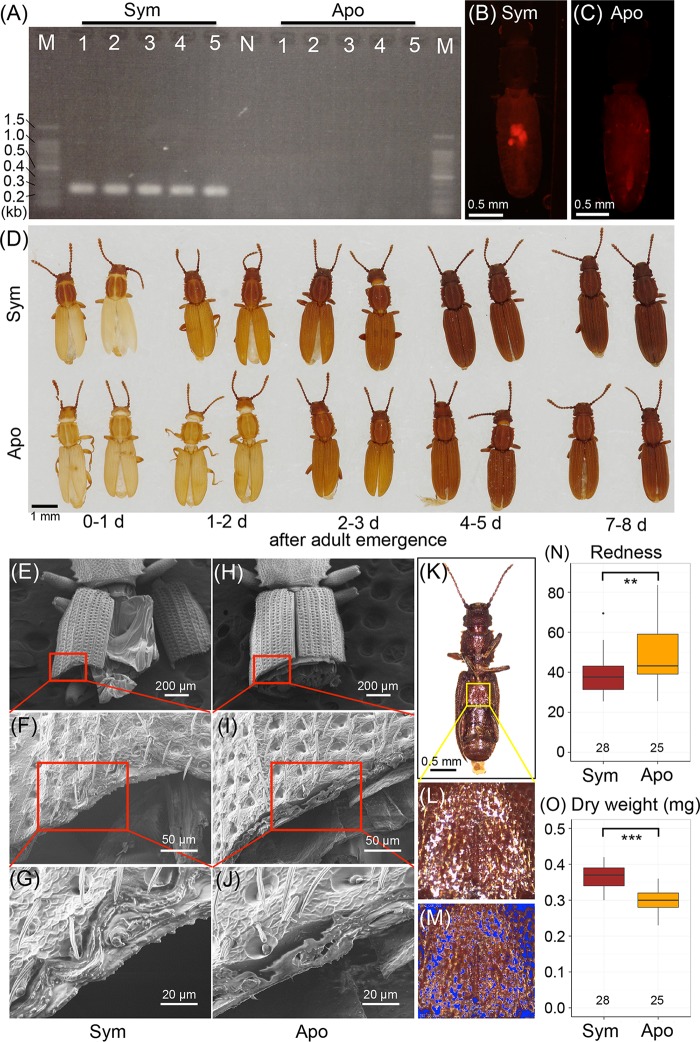
Effects of symbiont removal on adult phenotypes of *O. surinamensis*. (A) Diagnostic PCR detection of the symbiont from adult insects of the control symbiotic line (Sym) and the antibiotic-treated aposymbiotic line (Apo). Each lane in lanes 1 to 5 represents a DNA sample from a single insect. N and M represent the no-template control and DNA size markers, respectively. (B and C) Fluorescence *in situ* hybridization targeting 16S rRNA of the symbiont in adult females of the symbiotic strain (B) and the aposymbiotic strain (C). (D) Cuticle pigmentation after adult emergence of the symbiotic insects (upper row) and the aposymbiotic insects (lower row). (E to J) Scanning electron microscopic images of sectioned elytra of the symbiotic adult insects (E to G) and the aposymbiotic adult insects (H to J). Panels F and I are magnified images of panels E and H, as indicated by red rectangles; panels G and J are magnified images of panels F and I. (K to M) Process of quantifying redness of cuticle. From each of the ventral images of adult insects (K), a square area was extracted from the metasternum. On the square image (L), the pixels whose brightness was either above the top 10% or below the bottom 10% were masked in blue (M) and excluded from the analysis to minimize the effects of highlights and shadows. Then, RGB values for all (*n*) pixels were measured and averaged to obtain the redness index by Σ[*R* − mean (R, G, B)]/*n*. (N) Comparison of cuticle redness between the symbiotic insects and the aposymbiotic insects older than 2 weeks after adult emergence. (O) Comparison of dry body weight between the symbiotic insects and the aposymbiotic insects older than 2 weeks after adult emergence. In panels N and O, asterisks indicate statistically significant differences by *t* test: **, *P* < 0.01; ***, *P* < 0.001.

In an attempt to investigate functional aspects of the endosymbiotic association, we tried to remove the symbiont infection from *O. surinamensis* by antibiotic treatment. Adult insects from the same laboratory colony were divided into two groups, and the control group was fed with unsupplemented whole-wheat flour, while the antibiotic-treated group was fed with whole-wheat flour supplemented with 0.05% tetracycline. After the feeding treatment for two generations, newly emerged adult insects of each group were transferred to and maintained on unsupplemented whole-wheat flour, thereby creating a control line and an antibiotic-treated line of *O. surinamensis*. Diagnostic PCR revealed that the insects of the antibiotic-treated line were all symbiont negative, whereas the insects of the control line were all symbiont positive (Fig. 8A), verifying removal of the symbiont in the antibiotic-treated line. Fluorescence *in situ* hybridization confirmed absence of the symbiont in the bacteriomes of the antibiotic-treated insects (Fig. 8B and C). Interestingly, the insects of the antibiotic-treated line grew and reproduced normally with no difficulty in rearing and maintenance. Thus far, we have been stably maintaining both the symbiotic control line and the aposymbiotic antibiotic-treated line for over 20 months (Fig. 8A), indicating that the symbiont is not essential for normal growth, survival, and reproduction of *O. surinamensis*.

### Effects of symbiont removal on host insect phenotypes.

However, our close inspection of the symbiotic insects and the aposymbiotic insects unveiled subtle but remarkable phenotypic differences between them. Upon eclosion, the pronotum of the symbiotic adult insects was pigmented in brown, in contrast to the pale pronotum of the aposymbiotic adult insects (Fig. 8D, 0 to 1 day). The differences in cuticle pigmentation between the symbiotic insects and the aposymbiotic insects were the most prominent on the next day (Fig. 8D, 1 to 2 days), subsequently became less conspicuous as the insects aged and the cuticular pigmentation proceeded (Fig. 8D, 2 to 3, 4 to 5, and 7 to 8 days), and finally became almost indiscernible in mature adult insects that were older than 2 weeks and dark in color. Even in the mature adult insects, however, detailed analyses uncovered notable differences between the symbiotic insects and the aposymbiotic insects. Scanning electron microscopy revealed that sectioned elytra of the symbiotic insects tended to be structurally denser than the sectioned elytra of the aposymbiotic insects, although the differences were rather qualitative and varied depending on individuals (Fig. 8E to J). Color imaging analysis of the adult metasternum (Fig. 8K to M) demonstrated that the cuticle of the aposymbiotic insects was significantly more reddish than the cuticle of the symbiotic insects (Fig. 8N). Weighing of acetone-preserved and dried insects revealed that the aposymbiotic insects were slightly but significantly lighter in body weight than the symbiotic insects (Fig. 8O).

## DISCUSSION

Since the pioneering histological descriptions and some experimental studies dating back to the 1930s (45–47), we finally characterized the microbiological nature of the bacteriome-associated symbiont of the stored-product pest beetle, *O. surinamensis*, as a distinct bacterial lineage in the *Bacteroidetes* (Fig. 2). This study is the first to identify the bacteriome-associated endosymbiont from the beetle family Silvanidae.

Notably, the symbiont of *O. surinamensis* was the most closely related to the symbiont of *R. dominica*, forming a deeply diverged but statistically well-supported clade in the *Bacteroidetes* (Fig. 2). However, the phylogenetic affinity of the symbiotic bacteria is not concordant with the systematics of the host insects: the family Silvanidae is placed within the infraorder Cucujiformia, the family Bostrichidae belongs to the infraorder Bostrichiformia, and Cucujiformia is phylogenetically not allied to the Bostrichiformia in the Coleoptera (48). These phylogenetic patterns suggest the following possibilities: (i) a deep common ancestry of the symbiont originating from the common ancestor of the Cucujiformia and the Bostrichiformia, (ii) lateral symbiont transfer between the Silvanidae and the Bostrichidae, or (iii) independent symbiont acquisitions in the Silvanidae and the Bostrichidae from the common or closely related source bacteria belonging to the *Bacteroidetes*. Their common ecological aspect, living on cereal grains and other stored products, may have some relevance to these evolutionary patterns. A wider survey and molecular phylogenetic analyses of the bacteriome-associated endosymbionts in the Silvanidae and the Bostrichidae are needed to address which of these hypotheses is the most appropriate.

Judging from the AT-biased nucleotide composition (Fig. 2) and the accelerated molecular evolution (Table 1), it seems likely that the symbiont of *O. surinamensis* has experienced reductive genome evolution like the other endosymbiont lineages in the *Bacteroidetes*, like *Sulcia* (0.19 to 0.29 Mb) (49–51), *Blattabacterium* (0.59 to 0.64 Mb) (52–54), *Uzinura* (0.26 Mb) (55), and *Walczuchella* (0.31 Mb) (56). Although speculative, the extremely elongated cell shape of the symbiont of *O. surinamensis* may be relevant to the degenerative genome evolution and resultant deficiency in bacterial cell division and/or cell wall formation (57). Plausibly, the strange rosette-like cell shape of the phylogenetically related symbiont of *R. dominica* might be also accounted for in this context (42). Symbiont genome sequencing will provide pivotal clues to understanding these evolutionary issues.

A notable aspect of the endosymbiosis in *O. surinamensis* is that, although possessing the well-developed bacteriomes, the host insect is able to survive, grow, and reproduce even in the absence of the symbiont. This observation agrees with the following early experimental studies on *O. surinamensis*. Koch (46) established aposymbiotic colonies of *O. surinamensis* by high-temperature treatments at 36 to 38°C and reported that (i) the aposymbiotic insects were easily maintainable for as long as 5 years, (ii) during the period equivalent to some 25 insect generations, the aposymbiotic insects continued to form sterile bacteriomes in the abdomen, and (iii) no differences in growth and fecundity were detected between the aposymbiotic insects and the original symbiotic insects on a variety of foods, including oats, oatmeal, wheat, barley, buckwheat, hazelnut, and dried banana. Huger (47) reported that (i) antibiotic treatments also yielded aposymbiotic insects of *O. surinamensis*, (ii) a low-temperature treatment at 4°C for 24 days reduced the symbiont titer but did not eradicate it, and (iii) 45% of *O. surinamensis* strains collected from heavily infested grain warehouses turned out to be aposymbiotic, which might be attributable to overheating caused by massive proliferation of the insects in the closed grain stockrooms.

However, we strongly suspect that detailed fitness monitoring under a variety of environmental conditions, such as different diets, different nutritional conditions, different temperatures, different levels of humidity, with/without predators/parasites, etc., would lead to identification of ecological factors responsible for the persistence of the symbiont in *O. surinamensis* populations despite the dispensable nature of the symbiosis. In this context, our discovery of the slight but significant effects of aposymbiosis on the cuticle color and structure (Fig. 8) seems to be of importance. The sclerotized cuticle of beetles has been shown to confer mechanical strength, antipredator defense, tolerance to desiccation, and other beneficial consequences, which contribute to their survival and adaptation (58–61). Rearing experiments under these different conditions and strict fitness measurements are currently in progress to test these possibilities.

Previous studies on a variety of symbiont-dependent insects documented that experimental symbiont suppression often induced frail host insects with a pale and soft cuticle, as in, for example, aphids (62, 63), stinkbugs (15, 64–66), weevils (67–71), and others. These phenotypes may be due to general physiological or nutritional deficiency due to aposymbiosis (72, 73) or more specifically may be attributable to deficiency in symbiont-provisioned aromatic amino acids, namely, phenylalanine and tyrosine, which are the principal precursors needed for cuticle formation and hardening, as has been demonstrated in aphids (74, 75) and weevils (68, 70, 71). Genomic, transcriptomic, and metabolic analyses of the bacteriomes of *O. surinamensis* will provide pivotal clues to understanding what molecular and physiological mechanisms of the symbiont are involved in the cuticle formation of the host insect.

Finally, we point out that the easy rearing system for *O. surinamensis*, solely on whole-wheat flour in plastic containers almost free of laborious maintenance, in combination with the experimental tractability of both the symbiotic and aposymbiotic insects will facilitate experimental studies on nutritional, physiological, and metabolic aspects of host-symbiont interactions. In the flour beetle *Tribolium castaneum*, which is also maintainable on whole-wheat flour and renowned as a model insect in population biology, molecular genetics, and genomics (76–78), a nearly completely defined diet consisting of casein, glucose, cholesterol, vitamins, minerals, salts, and the insoluble fraction of yeast was developed for strict nutritional studies (79, 80). Development of a similar defined diet for *O. surinamensis* would enable detailed studies on functional aspects of the endosymbiosis by experimentally manipulating the diet components and by tracing the metabolites at the host-symbiont interface.

## MATERIALS AND METHODS

### Insects and rearing.

We mainly used a long-lasting laboratory strain of *O. surinamensis*, OsNFRI, which is of unknown origin, has been maintained for over 20 years at the National Food Research Institute, Tsukuba, Japan, and was provided by Akihiro Miyanoshita. The insects were reared on whole flour of domestically grown wheat (Ebetsu Flour Mill, Hokkaido, Japan) in plastic containers at 25°C under constant darkness. For symbiont elimination, the insects were reared on the whole-wheat flour supplemented with 0.05% tetracycline for two generations, and subsequently newly emerged adult insects were transferred to and maintained on the whole-wheat flour without the antibiotic. We also analyzed a strain of *O. surinamensis* provided by Kohjiro Tanaka, which was collected from sunflower seeds and maintained at Fuji Environment Service (Shizuoka, Japan), and a sample of *O. surinamensis* collected on 2 July 2017 at Ojiya, Niigata, Japan. Some insects were preserved in acetone as described previously (81) until DNA and phenotypic analyses.

### DNA analysis.

The adult abdomens were dissected and subjected to DNA extraction using a QIAamp DNA minikit (Qiagen). A 1.5-kb segment of bacterial 16S rRNA gene was amplified by PCR with the primers 10FF (5′-AGT TTG ATC ATG GCT CAG GAT-3′) and 1515R (5′-GTA CGG CTA CCT TGT TAC GAC TTA G-3′) (14) under the temperature profile of 94°C for 5 min followed by 35 cycles of 94°C for 30 s, 55°C for 30 s, and 72°C for 1 min and a final incubation at 72°C for 5 min. The PCR products were cloned and sequenced as described previously (82). Diagnostic PCR detection of the symbiont was conducted with the primers OsFlav60F (5′-AAG TCG AGG GGC AAC ATG AA-3′) and OsFlav304R (5′-CTC AGG CCC CCT ACC GAT AA-3′) specifically targeting a 0.3-kb segment of 16S rRNA gene of the symbiont under the same temperature profile. The PCR products were electrophoresed in 1.5% agarose gels, stained with ethidium bromide, and photographed on a UV transilluminator.

### Molecular phylogenetic and evolutionary analyses.

Multiple alignments of nucleotide sequences were constructed by ClustalW (83) implemented in MEGA v7.0.26 (84). The alignments were inspected and corrected manually and subjected to neighbor-joining analyses by MEGA v7.0.26 (84) with 1,000 bootstrap replicates, maximum likelihood analyses by MEGA v7.0.26 (84) with 1,000 bootstrap replicates, and Bayesian analyses by MrBayes v3.2.6 (85). The best-fit substitution model for the aligned sequences was evaluated by Kakusan v4 (86), which selected the generalized time-reversible (GTR) gamma model for both the maximum likelihood and Bayesian methods. Relative rate tests were performed by RRTree (87).

### Histological procedures.

For observation of fresh cell images of the symbiont, the bacteriomes were dissected in phosphate-buffered saline (PBS: 0.8% NaCl, 0.02% KCl, 0.115% Na_2_HPO_4_, 0.02% KH_2_PO_4_ [wt/vol]), placed on a glass slide with a drop of SYTOX Green solution (1/1,000 dilution) or 4 μM 4′,6-diamidino-2-phenylindole solution, smeared with a coverslip, and observed under an epifluorescence microscope (Axiophot; Zeiss). For whole-mount fluorescence *in situ* hybridization, adults, pupae, larvae, and eggs were pretreated in 70% ethanol under a dissection microscope as follows. Adults were deprived of all wings by forceps, and a side of their abdomen was cut with a razor. Pupae and larvae were punctured by a needle at the anterior and posterior regions of the body. Eggs containing embryos were collected, treated with 50% bleach for 2 min, and washed thoroughly with distilled water. The pupal and larval samples were fixed in Carnoy’s solution (60% ethanol, 30% chloroform, and 10% acetic acid [vol/vol]) for 3 h at room temperature, washed thoroughly with 70% ethanol, treated with 6% H_2_O_2_ in 80% ethanol for 6 days to quench autofluorescence of the tissues (88), and washed thoroughly with 70% ethanol. The adult and egg samples were fixed with 4% formalin in PBS and washed thoroughly with PBS. For fluorescence *in situ* hybridization of dissected bacteriomes, adults, pupae, and larvae were dissected in 4% formalin in PBS, and the dissected tissues were fixed in fresh 4% formalin in PBS for 1 h at room temperature and washed thoroughly with PBS.

### Fluorescence *in situ* hybridization.

Fluorescence *in situ* hybridization targeting 16S rRNA of the symbiont was conducted essentially as described previously (88). The samples were rehydrated with PBT (PBS containing 0.1% Tween 20 [vol/vol]) and incubated in hybridization buffer (20 mM Tris-HCl [pH 8.0], 0.9 M NaCl, 0.01% sodium dodecyl sulfate, 30% formamide [wt/vol]) containing 100 pmol/ml each probe at room temperature overnight. The following probes whose 5′ end was labeled with Alexa Fluor 555 were used either singly or in combination: EUB338 (5′-GCT GCC TCC CGT AGG AGT-3′), targeting diverse bacteria (89); EUB897 (5′-TTT GAG TTT YAV YCT TGC G-3′), targeting diverse bacteria designed in this study; Sul664R (5′-CCM CAC ATT CCA GYT ACT CC-3′) targeting *Sulcia* and allied bacteria of the *Bacteroidetes* (90); and OSFlav1256R (5′-ATC TAA TTA CTT AGT AGC AGC C-3′), specifically targeting the symbiont of *O. surinamensis* designed in this study. After being washed with PBT three times for 10 min each at room temperature, the samples were placed on glass slides, mounted in 80% glycerol or SlowFade antifade reagent (Thermo Fisher Scientific), and observed under a fluorescence dissection microscope (M165FC; Leica), an epifluorescence microscope (Axiophot; Zeiss), and/or a laser scanning confocal microscope (LSM710; Zeiss). Digital images were merged and adjusted manually using Adobe Photoshop CC 2017 (Adobe).

### Electron microscopy.

For transmission electron microscopy, pupae were dissected in 2.5% glutaraldehyde in 0.1 M phosphate buffer (pH 7.4), and the dissected bacteriomes were prefixed with the fixative at 4°C overnight. Subsequently, the samples were postfixed with 2% osmium tetroxide in 0.1 M phosphate buffer (pH 7.4) at 4°C for 60 min, dehydrated through a water-ethanol series, and embedded in Epon 812 resin. Ultrathin sections (around 80 nm thick) were made by an ultramicrotome (EM UC7; Leica), mounted on copper meshes, stained with uranyl acetate and lead citrate, and observed under a transmission electron microscope (H-7600; Hitachi). For scanning electron microscopy, dry specimens of adult insects were cut with a razor blade. Subsequently, the samples were gold coated using Smart Coater (JEOL) and observed under a scanning electron microscope (JCM 6000; JEOL) operated at high vacuum and at 5 kV with use of the secondary electron signal.

### Qualitative and quantitative analyses of adult phenotypes.

From mass-cultured symbiotic and aposymbiotic colonies of *O. surinamensis*, all adult insects were removed and transferred to new rearing containers. Then, newly emerged adult insects were collected from the old rearing containers every day, by which we obtained age-defined symbiotic and aposymbiotic adult insects. The age-defined adult insects were preserved in acetone, dried in air, placed on a piece of double-sided adhesive tape, and photographed by a digital camera. Symbiotic and aposymbiotic mature adult insects older than 2 weeks after emergence were dehydrated in acetone for several days, dried in air for a day, and weighed on a microbalance (MT5; Mettler Toledo, Columbus, OH). Subsequently, the adult insects were photographed from the ventral side by a digital camera (EC3; Leica) connected to a dissection microscope (S8APO; Leica) under a constant-lighting condition. Image processing operations and measurements were carried out with the software Natsumushi v0.99 (https://sites.google.com/site/mtahashilucanid/program/natsumushi). From each image of the metasternum (Fig. 8K), a square area of maximal size was extracted (Fig. 8L). On the square image, the pixels whose brightness was either over the top 10% or below the bottom 10% were masked in blue and excluded from the analysis to minimize the effects of highlights and shadows (Fig. 8M). Then, 8-bit-integer RGB (red, green, blue) values (0 ≦ R, G, B ≦ 255) for all (*n*) pixels were measured and averaged to obtain the redness index by Σ[*R* − mean (R, G, B)]/*n*.

### Accession number(s).

The 16S rRNA gene sequences of the symbiont of *O. surinamensis* determined in this study have been deposited in the DNA Data Bank of Japan database under accession no. LC314798 to LC314800.
